# Yoga Therapy and Polyvagal Theory: The Convergence of Traditional Wisdom and Contemporary Neuroscience for Self-Regulation and Resilience

**DOI:** 10.3389/fnhum.2018.00067

**Published:** 2018-02-27

**Authors:** Marlysa B. Sullivan, Matt Erb, Laura Schmalzl, Steffany Moonaz, Jessica Noggle Taylor, Stephen W. Porges

**Affiliations:** ^1^Integrative Health Sciences, Maryland University of Integrative Health, Laurel, MD, United States; ^2^The Center for Mind-Body Medicine, Washington, DC, United States; ^3^College of Science and Integrative Health, Southern California University of Health Sciences, Whittier, CA, United States; ^4^Director of Clinical and Academic Research, Maryland University of Integrative Health, Laurel, MD, United States; ^5^Independent Researcher, Decatur, GA, United States; ^6^Kinsey Institute, Indiana University, Bloomington, IN, United States; ^7^Department of Psychiatry, University of North Carolina at Chapel Hill, Chapel Hill, NC, United States

**Keywords:** yoga therapy, Polyvagal Theory, self-regulation, resilience, vagus nerve, interoception, stress response, allostatic load

## Abstract

Yoga therapy is a newly emerging, self-regulating complementary and integrative healthcare (CIH) practice. It is growing in its professionalization, recognition and utilization with a demonstrated commitment to setting practice standards, educational and accreditation standards, and promoting research to support its efficacy for various populations and conditions. However, heterogeneity of practice, poor reporting standards, and lack of a broadly accepted understanding of the neurophysiological mechanisms involved in yoga therapy limits the structuring of testable hypotheses and clinical applications. Current proposed frameworks of yoga-based practices focus on the integration of bottom-up neurophysiological and top-down neurocognitive mechanisms. In addition, it has been proposed that phenomenology and first person ethical inquiry can provide a lens through which yoga therapy is viewed as a process that contributes towards eudaimonic well-being in the experience of pain, illness or disability. In this article we build on these frameworks, and propose a model of yoga therapy that converges with Polyvagal Theory (PVT). PVT links the evolution of the autonomic nervous system to the emergence of prosocial behaviors and posits that the neural platforms supporting social behavior are involved in maintaining health, growth and restoration. This explanatory model which connects neurophysiological patterns of autonomic regulation and expression of emotional and social behavior, is increasingly utilized as a framework for understanding human behavior, stress and illness. Specifically, we describe how PVT can be conceptualized as a neurophysiological counterpart to the yogic concept of the *gunas*, or qualities of nature. Similar to the neural platforms described in PVT, the *gunas* provide the foundation from which behavioral, emotional and physical attributes emerge. We describe how these two different yet analogous frameworks—one based in neurophysiology and the other in an ancient wisdom tradition—highlight yoga therapy’s promotion of physical, mental and social wellbeing for self-regulation and resilience. This parallel between the neural platforms of PVT and the *gunas* of yoga is instrumental in creating a translational framework for yoga therapy to align with its philosophical foundations. Consequently, yoga therapy can operate as a distinct practice rather than fitting into an outside model for its utilization in research and clinical contexts.

## Introduction

Mind-body therapies, including yoga therapy, are proposed to benefit health and well-being through an integration of top-down and bottom-up processes facilitating bidirectional communication between the brain and body (Taylor et al., 2010; Muehsam et al., 2017). Top-down processes, such as the regulation of attention and setting of intention, have been shown to decrease psychological stress as well as hypothalamic-pituitary axis (HPA) and sympathetic nervous system (SNS) activity, and in turn modulate immune function and inflammation (Taylor et al., 2010; Muehsam et al., 2017). Bottom-up processes, promoted by breathing techniques and movement practices, have been shown to influence the musculoskeletal, cardiovascular and nervous system function and also affect HPA and SNS activity with concomitant changes in immune function and emotional well-being (Taylor et al., 2010; Muehsam et al., 2017).

The top-down and bottom-up processes employed in mind-body therapies may regulate autonomic, neuroendocrine, emotional and behavioral activation and support an individual’s response to challenges (Taylor et al., 2010). Self-regulation, a conscious ability to maintain stability of the system by managing or altering responses to threat or adversity, may reduce symptoms of diverse conditions such as irritable bowel syndrome, neurodegenerative conditions, chronic pain, depression and PTSD through the mitigation of allostatic load with an accompanying shift in autonomic state (Taylor et al., 2010; Streeter et al., 2012; Gard et al., 2014; Schmalzl et al., 2015; Muehsam et al., 2017). Gard et al. (2014) have proposed such a model of top-down and bottom-up self-regulatory mechanisms of yoga for psychological health.

Resilience may provide another benefit of mind-body therapies as it includes the ability of an individual to “bounce back” and adapt in response to adversity and/or stressful circumstances in a timely way such that psychophysiological resources are conserved (Tugade and Fredrickson, 2004; Resnick et al., 2011; Haase et al., 2016; Whitson et al., 2016). High resilience is correlated with quicker cardiovascular recovery following subjective emotional experiences (Tugade and Fredrickson, 2007), less perceived stress, greater recovery from illness or trauma and better management of dementia and chronic pain (Resnick et al., 2011). Compromised resilience is linked to dysregulation of the autonomic nervous system through measures of vagal regulation (respiratory sinus arrhythmia) (Dale et al., 2009). Yoga is correlated with both improvement in measures of psychological resilience (Dale et al., 2011) and improved vagal regulation (Sarang and Telles, 2006; Khattab et al., 2007; Telles et al., 2016; Tyagi and Cohen, 2016; Chu et al., 2017).

This article explores the integration of top-down and bottom-up processes for self-regulation and resilience through both Polyvagal Theory (PVT; Porges, 2011) and yoga therapy. PVT will be described in relation to contemporary understandings of interoception as well as the biobehavioral theory of the “preparatory set”, which will be defined later. This will help to lay out an integrated systems view from which mind-body therapies facilitate the emergence of physiological, emotional and behavioral characteristics for the promotion of self-regulation and resilience.

**Figure 1:**
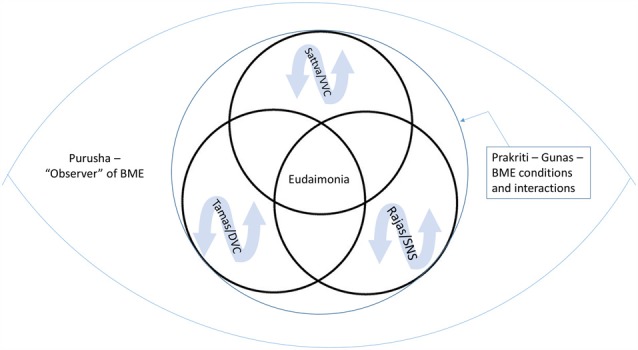
The central eye represents the body, mind and environmental context (BME) and the peripheral eye represents the context of an observer/experiencer of that content. Within Prakriti, resilience is represented by the capacity to recognize and shift states, as well as changing the relationship to the fluctuations of the *gunas* (rajas/tamas/sattva) and neural platforms (sympathetic nervous system (SNS)/dorsal vagal complex (DVC)/ventral vagal complex (VVC)). Yoga aims to facilitate the emergence of qualities such as eudaimonia by strengthening the experience of sattva and VVC as well as developing facility in moving between *gunas* and neural platforms and changing the relationship and response to the inherent changing nature of the body, mind and environment reflected in *gunas* and neural platforms.

We will examine the convergence of the neural platforms, described in PVT, with the three *gunas*, a foundational concept of yogic philosophy that describes the qualities of material nature. Both PVT and yoga provide frameworks for understanding how underlying neural platforms (PVT) and *gunas* (yoga) link the emergence and connectivity between physiological, psychological and behavioral attributes. By affecting the neural platform, or *guna* predominance, as well as one’s relationship to the continual shifting of these neural platforms, or *gunas*, the individual learns skills for self-regulation and resilience. Moreover these frameworks share characteristics that parallel one another where the neural platform reflects the *guna* predominance and the *guna* predominance reflects the neural platform (see Figure 1).

This exploration is intended to be a comparative and translatory approach aimed at enabling the complexity of the yoga tradition to be understood for its benefits and application into modern healthcare contexts, while still rooted in its own traditional wisdom and explanatory framework. A model through which self-regulation and resilience occurs is described from a yoga foundational framework which converges with current ideas in neurophysiology and biobehavioral regulation (see Figure 1).

## Polyvagal Theory (PVT)

PVT, and other emerging theories such as neurovisceral integration (Thayer and Lane, 2000; Smith et al., 2017), help elucidate connections between the systems of the body, the brain, and the processes of the mind offering increased insight into complex patterns of integrated top-down and bottom-up processes that are inherent to mind-body therapies. PVT delineates three distinct neural platforms in response to perceived risk (i.e., safety, danger, life-threat) in the environment that operate in a phylogenetically determined hierarchy consistent with the Jacksonian principle of dissolution (Jackson, 1884; Porges, 2001, 2003). PVT introduces the concept of neuroception to describe the subconscious detection of safety or danger in the environment through bottom-up processes involving vagal afferents, sensory input related to external challenges, and endocrine mechanisms that detect and evaluate environmental risk prior to the conscious elaboration by higher brain centers (Porges, 2003).

The three polyvagal neural platforms, as described below, are linked to the behaviors of social communication, defensive strategy of mobilization and defensive immobilization (Porges, 1995, 1998, 2001, 2003, 2007, 2009, 2011):

1. The ventral vagal complex (VVC) provides the neural structures that mediate the “social engagement system”. When safety is detected in the internal and external environment, the VVC provides a neural platform to support prosocial behavior and social connection by linking the neural regulation of visceral states supporting homeostasis and restoration to facial expressivity and the receptive and expressive domains of communication (e.g., prosodic vocalizations and enhanced ability to listen to voice). The motor component of the VVC, which originates in the nucleus ambiguus (NA), regulates and coordinates the muscles of the face and head with the bronchi and heart. These connections help orient the person towards human connection and engagement in prosocial interactions and provide more flexible and adaptive responses to environmental challenges including social interactions (Porges, 2011, 2017; Porges and Carter, 2017).
2. The SNS is frequently associated with fight/flight behaviors. Fight/flight behaviors require activation of the SNS and are the initial and primary defense strategies recruited by mammals. This defense strategy requires increased metabolic output to support mobilization behaviors. Within PVT the recruitment of SNS in defense follows the Jacksonian principle of dissolution and reflects the adaptive reactions of a phylogenetically ordered response hierarchically in which the VVC has failed to mitigate threat. When the SNS circuit is recruited there are massive physiological changes including an increase in muscle tone, shunting of blood from the periphery, inhibition of gastrointestinal function, a dilation of the bronchi, increases in heart rate and respiratory rate, and a release of catecholamines. This mobilization of physiological resources sets the stage for responding to real or assumed danger in the environment and towards the end-goals of safety and survival. When the SNS becomes the dominant neural platform, the VVC influence may be inhibited in favor of mobilizing resources for rapid action. Whereas prosocial behaviors and social connection are associated with the VVC, the SNS is associated with behaviors and emotions such as fear or anger that help to orient to the environment for protection or safety.
3. The dorsal vagal complex (DVC) arises from the dorsal nucleus of the vagus (DNX) and provides the primary vagal motor fibers to organs located below the diaphragm. This circuit is designed to adaptably respond to immense danger or terror and is the most primitive (i.e., evolutionarily oldest) response to stress. Activation of the DVC in defense results in a passive response characterized by decreased muscle tone, dramatic reduction of cardiac output to reserve metabolic resources, alteration in bowel and bladder function via reflexive defecation and urination to reduce metabolic demands required by digestion and other bodily processes. This inhibition of viscera reflects an attempt to reduce metabolic and oxygen demands to the least amount necessary for survival. Behaviorally this is often referred to as immobilization or shutdown associated with feigning death, behavioral shutdown, collapse, or “freeze” responses, and may be experienced in humans as a disembodied dissociative state that may include loss of consciousness.

PVT posits that through these neural platforms particular physiological states, psychological attributes, and social processes are connected, emerge, and are made accessible to the individual (Porges, 1998, 2003, 2011, 2017; Porges and Carter, 2017). The physiological state established by these neural platforms in response to threat or safety (as determined via the integrated processes of neuroception) allows for or limits the range of emotional and behavioral characteristics that are accessible to the individual (Porges, 2003).

A core aspect of PVT is that patterns of physiological state, emotion and behavior are particular to each neural platform (for a detailed review of the neurophysiological, neuroanatomical, and evolutionary biological bases of PVT see Porges, 1995, 1998, 2001, 2007, 2009, 2011). For example, the neural platform of the VVC is proposed to connect visceral homeostasis with emotional characteristics and prosocial behaviors that are incompatible with the neurophysiological states, emotional characteristics or social behaviors that manifest in the neural platforms of defensive strategies seen in SNS or DVC activation. When the VVC is dominant, the vagal brake is implemented and prosocial behaviors and emotional states such as connection and love have increased potential to emerge. When the SNS is the primary defensive strategy, the NA turns off the inhibitory action of the ventral vagal pathway to the heart to enable sympathetic activation and behavioral and emotional strategies of mobilization are supported. If the DVC immobility response is the defensive strategy, the dorsal motor nucleus is activated as a protective mechanism from pain or potential death and active response strategies are not available (Porges, 1998, 2003, 2009, 2011; Porges et al., 2008; Porges and Carter, 2017). It is important to note the VVC has other attributes that enable blended states with the SNS (e.g., play) or with the DVC (e.g., intimacy). However, in these examples of blended states, the VVC remains easily accessible and functionally contains the subordinate circuits. When the VVC is functionally withdrawn it promotes accessibility of the SNS as a defense fight/flight system. Similarly, the SNS functionally inhibits access to the DVC immobilization shutdown response. Thus, the profound shutdown reactions that may lead to death becomes neurophysiologically accessible only when the SNS is reflexively inhibited.

## Vagal Activity, Interoception, Regulation and Resilience

Vagal activity, via ventral vagal pathways, is suggested to be reflective of regulation and resilience of the system where high cardiac vagal tone correlates with more adaptive top-down and bottom-up processes such as: attention regulation, affective processing and flexibility of physiological systems to adapt and respond to the environment (Thayer and Lane, 2000; Porges, 2011; Streeter et al., 2012; Park and Thayer, 2014; Strigo and Craig, 2016). Vagal control has also been shown to correlate with differential activation in brain regions that regulate responses to threat appraisal, interoception, emotion regulation, and the promotion of greater flexibility in response to challenge (Streeter et al., 2012; Park and Thayer, 2014). Conversely, low vagal regulation has been associated with maladaptive bottom-up and top-down processing resulting in poor self-regulation, less behavioral flexibility, depression, generalized anxiety disorder, and adverse health outcomes including increased mortality in conditions such as lupus, rheumatoid arthritis and trauma (Tsuji et al., 1994; Thayer and Lane, 2000; Park and Thayer, 2014; Muehsam et al., 2017).

The vagus nerve is comprised of 80% afferent fibers and serves as an important conduit for interoceptive communication about the state of the viscera and internal milieu to brain structures (Porges, 2004, 2011). Interoception has been explored as essential to the bridging of top-down and bottom-up processing and in the investigation of the relationships between sensations, emotions, feelings and sympathovagal balance (Porges, 1993; Craig, 2015; Farb et al., 2015; Strigo and Craig, 2016). Support has been found for the integration of interoceptive input, emotion and regulation of sympathovagal balance in the insular and cingulate cortices, facilitating a unified response of the individual to body, mind or environmental (BME) phenomena (Craig, 2015; Strigo and Craig, 2016).

Self-regulation is proposed to be dependent on the accuracy with which we interpret and respond to interoceptive information, with greater accuracy leading to enhanced adaptability and self-regulation (Farb et al., 2015). As such, interoception is considered to be important in pain, addiction, emotional regulation, and healthy adaptive behaviors including social engagement (Porges, 2011; Farb et al., 2015; Ceunen et al., 2016). In addition, interoception has been proposed as key to resilience as the accurate processing of internal bodily states promotes a quick restoration of homeostatic balance (Haase et al., 2016).

It has been proposed that mind-body therapies are an effective tool for the regulation of vagal function, with consequent fostering of adaptive functions including the mitigation of adverse effects associated with social adversity (Black et al., 2013; Cole, 2013; Bower et al., 2014), the reduction of allostatic load, and the facilitation of self-regulatory skills and resilience of the ANS across various patient populations and conditions (Streeter et al., 2012; Schmalzl et al., 2015; Muehsam et al., 2017; Porges, 2017; Porges and Carter, 2017).

## Polyvagal Theory and Mind-Body Therapies for Regulation and Resilience

Mind-body therapies emphasize the cultivation of somatic awareness, including both interoception and proprioception, combined with the mindfulness-based qualities of nonjudgment, non-reactivity, curiosity, or acceptance in order to engage in a process of re-appraisal of stimuli (Mehling et al., 2011; Farb et al., 2015). While being encouraged to cultivate awareness of BME phenomena and stimuli, the individual is supported in a process of re-interpretation or re-orientation to such stimuli so that insight may occur and adaptability, regulation, and resilience may be fostered (Mehling et al., 2011; Farb et al., 2015). This capacity to alter the relationship and reaction to BME phenomena is thought to be essential for self-regulation and well-being (Farb et al., 2015). It has been shown that patients utilizing mind-body therapies for healing reported both a shift in their experience and response to negative emotions and sensations as well as the development of self-regulatory skills in dealing with pain, emotional regulation and re-appraisal of life situations (Mehling et al., 2011).

PVT offers insight into how learning to recognize and shift the underlying neural platform of any given psychophysiological state, may directly affect physiology, emotion and behavior thus helping the individual cultivate adaptive strategies for regulation and resilience to benefit physical, mental and social health (Porges, 2011). As mind-body therapies affect the vagal pathways they are suggested to form a means of “exercising” these neural platforms to foster self-regulation and resilience of physiological function, emotion regulation and prosocial behaviors (Gard et al., 2014; Schmalzl et al., 2015; Porges, 2017; Porges and Carter, 2017).

Optimal neural regulation of the autonomic nervous system and the related endocrine and immune systems is fostered through active engagement of the VVC by utilizing specific movements or positions, breathing practices, chanting or meditation which affects both top-down and bottom-up processes (Cottingham et al., 1988a,b; Eckberg, 2003; Hayano and Yasuma, 2003; Porges, 2017; Porges and Carter, 2017). Resilience is proposed to be fostered by both downregulating defensive states and supporting more flexibility and adaptability in relationship to various phenomena of the BME to promote physiological restoration as well as positive psychological and social states (Porges, 2017; Porges and Carter, 2017). The individual can learn to improve activation of the VVC with its homeostatic influence on the organism, as well as increase the facility to move in and out of other neural platforms such as the SNS or DVC when real or perceived stress is encountered.

In sum, mind-body practices can teach the individual to make the VVC more accessible, widen the threshold of tolerance to other neural platforms, change the relationship and response to SNS and DVC neural platforms that occur as natural fluctuations of the BME, and how to become more skilled at moving in and out of these neural platforms (Porges, 2017; Porges and Carter, 2017). Breathing maneuvers within yoga often facilitate similar shifts in autonomic state with convergent psychological and health consequences (e.g., Brown and Gerbarg, 2005a,b, 2012; Brown et al., 2013). These practices may also contribute to our potential to experience connection beyond social interactions or networks and to a more universal and unbounded sense of oneness and connection (Porges, 2017).

## Five Global States and Preparatory Sets

PVT further proposes that the three neural circuits of SNS, VVC and DVC are not mutually exclusive nor antagonistic, rather these three circuits co-arise, co-exist and co-mingle to create the array of complex human physiological, emotional and behavioral states (Porges, 1998, 2011). Bernston elucidated co-activation and complexity in SNS and PNS interactions in the doctrine of autonomic space (Berntson et al., 1991). This complexity allows for response to threat to start with a withdrawal of cardiac vagal tone before SNS activation as well as greater flexibility and precision to adjust to circumstances.

The PVT defines five global states based on the neural platform(s) which are predominant or active (Porges, 1998, 2011). The VVC, SNS and DVC circuits as just described represent three of the global states, and the other two arise from their co-activation.

When the VVC and SNS circuits co-arise there is a fourth state of safe mobilization. The VVC enables the experience of safety and connection, while the SNS supports the mobilization of the body’s resources for dexterity, movement, and the quick or creative thinking needed for activities such as dance, play, artistic expression, or writing. Mind-body practices such as the postural practice of hatha yoga or tai chi are examples where the body can be mobilized for action, but the mind and breath provide the stimulus for calmness, safety and connection.

The fifth state arises from the co-activation of the VVC and DVC. These two circuits working together facilitate the state of safe immobilization. Immobilization without fear allows for the emergence of social bonds to be formed through prosocial activities such as childbirth, conception and nursing.

The concept of preparatory sets provides for a dynamic understanding of the relationship between physical posture/muscle tone, visceral state/ANS, affective state, arousal/attention and cognitive expectation (Payne and Crane-Godreau, 2015). A change to any one of these components will result in shifts throughout the preparatory set leading to an integrated reaction by the human system in response to the needs of the environment or situation.

The five global states of the PVT reflect a complexity of interactions throughout the preparatory set by way of the portal of the ANS resulting in corresponding changes along somatomotor, affective and cognitive levels (Payne and Crane-Godreau, 2015). In other words, by impacting the neural platforms from which an individual is operating, there are concomitant effects on muscle tone, visceral state, attention, affect and cognition.

Significant to this is the possibility that prolonged time in any PVT-defined maladaptive threat state may contribute to disorders or conditions which manifest with a combined disturbance of physiology, emotion and behavior (Porges and Kolacz, in press). Porges and Kolacz (in press) have suggested the plausibility of autonomic dysregulation as a causative factor in irritable bowel syndrome and fibromyalgia both of which are characterized by altered physiology including decreased cardiac vagal tone inferred from a lack of heart rate variability (HRV), absence of obvious tissue pathology, and oftentimes associated with a history of trauma (Kolacz and Porges, in press). This view is consistent with the expanding body of research linked to the original CDC-Kaiser Adverse Childhood Experiences Study demonstrating strong correlation of stress and trauma history to various pathologies later in life (Felitti et al., 1998).

An important insight that comes from an integrated understanding of PVT and preparatory sets is the necessity to investigate mind-body therapies as they were originally intended to be practiced, as integrative methodologies affecting simultaneous components of the individual’s experience (Payne and Crane-Godreau, 2015). Many mind-body therapies, including yoga, call for simultaneous attention to the body, breathing, attentional and affective regulation and cognition. Thereby representing a comprehensive methodology and set of practices integrating both top-down and bottom-up processes in response to BME phenomena. It is imperative to realize that when these mind-body systems are reduced to investigate just one component, they are being taken outside the original context of their cohesive practice, thereby likely diminishing the intended combined effect.

Mind-body practices teach the individual to become aware of their preparatory set, to effectively shift unhealthy patterns of response to BME stimuli within their preparatory set, and learn healthier and more adaptive preparatory set patterns in response to BME phenomena through various techniques (Payne and Crane-Godreau, 2015). Tools for self-regulation and the cultivation of resilience may occur as the individual learns the state of safe mobilization such that activation of the system does not drive out positive affective states or prosocial behavior and connection.

## Yoga and Yoga Therapy

Payne and Crane-Godreau suggest yoga as one mind-body practice that can shift the preparatory set (Payne and Crane-Godreau, 2015). Yoga consists of a variety of practices which may serve to affect one or more components of the preparatory set by influencing muscle tone/posture, ANS, attention, affect, or cognition (Payne and Crane-Godreau, 2015). Yoga practices can be utilized to affect the ANS and to both manipulate and change the relationship to these shifting neural platforms described in PVT.

While much of modern yoga practice focuses primarily on physical postures and movement sequences, the traditional roots are centered on a philosophical path towards understanding the causes of suffering and its alleviation (Stoler-Miller, 1998, 2004; Easwaran, 2007; Singleton, 2010; Miller, 2012; Mallinson and Singleton, 2017). To this point, recent work by Mallinson and Singleton (2017) highlights the variable meaning of yoga throughout the ancient texts. Historically the word yoga has been used to describe both the method of prescribed sets of practices (yoga as methodology) and the aim or goal of these practices (yoga as a state of being; Mallinson and Singleton, 2017). Yoga as state of being includes the definition as “union” which can mean union with one’s own essential nature or a supreme Self (Mallinson and Singleton, 2017). Other definitions of yoga include equanimity and “skill in action” (Mallinson and Singleton, 2017). Due to the variable definitions of yoga and in an effort to be nonbiased to any one perspective, we will utilize concepts common throughout yogic texts such as the Upanishads (Easwaran, 2007), the Bhagavad Gita (Stoler-Miller, 2004), Samkhya Karika (Miller, 2012) and the Yoga Sutras (Stoler-Miller, 1998). By using concepts central to each of these texts, we will propose a framework that traverses individual lineages and helps create a shared language and understanding for yoga including relationship to current contexts.

Yoga therapy is an evolving practice in complementary and integrative healthcare (CIH) with recent accreditation for schools and credentialing of yoga therapists^1^. Yoga therapy is grounded in the ancient wisdom and practices of yoga, integrated with scientific knowledge for application into current healthcare contexts. There are a multiplicity of ways to define what constitutes a yoga practice. As such, an obstacle to the professionalization of yoga therapy has been reported as the heterogeneity of the practices and poor research reporting (Jeter et al., 2015). An explanatory framework for yoga therapy is therefore key to the understanding and utilization of yoga therapy as a unique and distinct CIH profession.

## Explanatory Framework for Yoga Therapy

Recent work has begun to define and establish an explanatory framework for yoga therapy and to suggest theoretical frameworks outlining the mechanisms underlying yoga-based practices from both neurophysiological and psychological perspectives (Streeter et al., 2012; Gard et al., 2014; Schmalzl et al., 2015; Sullivan et al., 2018). A recent explanatory model based on the philosophical and ethical foundations of yoga explored yoga therapy as a methodology for alleviating suffering by transforming an individual’s relationship to BME phenomena and catalyzing the emergence of eudaimonic wellbeing (Sullivan et al., 2018).

Eudaimonia represents a state of human flourishing or sense of well-being that is non-transitory and is often connected to a sense of meaning, purpose, or self-realization (Ostwald, 1962; Keyes and Simoes, 2012). Eudaimonic well-being is linked to many health benefits such as: the mitigation of gene expression changes in response to social adversity; reduction in perceived loneliness; decreased inflammation; improved immune regulation; mental flourishing; and decreased all-cause mortality independent of other variables (Keyes and Simoes, 2012; Fredrickson et al., 2013; Cole et al., 2015). Yoga has been correlated with both eudaimonia (Ivtzan and Papantoniou, 2014) and related gene expression changes found in the mitigation of the response to social adversity resulting in decreased inflammation and improved immune regulation (Black et al., 2013; Bower et al., 2014). As such, it could be hypothesized that yoga facilitates its many positive physiological, mental and social health benefits through its capacity to facilitate eudaimonic well-being. An explanatory framework of yoga therapy focused on its intention to promote the emergence of eudaimonic well-being with its concomitant physiological and mental health benefits is significant for both research and expanded integration into modern healthcare contexts for a wide variety of patient populations and conditions.

## Yoga’s Philosophical Foundation: *Prakriti* and *Purusha*

Yoga teaches that suffering arises from the individual’s relationship, reaction to, and misidentification with the various phenomena of the BME (Stoler-Miller, 1998, 2004; Miller, 2012). Yoga practices are intended to teach a method of discrimination to facilitate a change in the relationship to BME phenomena and ultimately in the experience of suffering itself (Stoler-Miller, 1998, 2004; Bawra, 2012; Miller, 2012). Through yoga, the individual learns both the patterns of behavior and actions, which may perpetuate their suffering as well as a path towards a shift in those patterns for the potential alleviation of suffering.

This process of discernment to move from suffering to a change in identification with such suffering and possibly its alleviation is taught through inquiry into the difference between material nature, termed *prakriti*, and spirit, termed *purusha* (Bawra, 2012; Miller, 2012). *Purusha* can be defined as spirit, the indweller, the observer, the seer or “that which sees” and is said to be the experiencer of material nature (Bawra, 2012; Miller, 2012). *Prakriti* is the term given to all of material nature, or all that is seen, changes, and is made manifest (Bawra, 2012; Miller, 2012; Mallinson and Singleton, 2017). The clarity that arises from this discrimination and realization of the difference between *purusha* and *prakriti* shifts the yoga practitioner’s relationship to BME phenomena such that suffering is eased and the experience of steadfast joy, or eudaimonia, may potentially emerge (Stoler-Miller, 1998, 2004; Easwaran, 2007; Bawra, 2012; Miller, 2012).

## The *Gunas*, Qualities of Material Nature

*Prakriti* is said to be comprised of three qualities. These three qualities, named the *gunas*, are said to underlie and shape the characteristics of everything that is of material nature including the BME (Bawra, 2012; Miller, 2012). The *gunas* enable and support a dynamism to the BME in which the fluctuations and differing proportions of these qualities give everything in the BME their unique and varied characteristics (Stoler-Miller, 2004; Bawra, 2012; Miller, 2012). As mentioned earlier, the root of suffering is said to arise from the misidentification of *Purusha* with the various phenomena of the BME, or *Prakriti*, and more specifically with the *gunas* (Stoler-Miller, 1998, 2004; Bawra, 2012; Miller, 2012). The knowledge and practices of yoga are intended to assist the individual in the realization that they may be “experiencing” the *gunas* vs. “being” the *gunas*. The apprehension and discernment of these three *gunas* is key to the realization of the difference between *purusha* and *prakriti*; thereby offering insight into the causes of suffering as well as its alleviation.

The Samkhya Karika, a text representing a seminal philosophy found across the yoga tradition, as well as the Bhagavad Gita and the Yoga Sutras of Patanjali describes the *gunas* and their emergent physical, mental and behavioral attributes as follows (Stoler-Miller, 1998, 2004; Bawra, 2012; Miller, 2012; Larson and Īśvarakrsna, 2014).

*Sattva* is the quality of pleasure, calmness and tranquility that serves the function of illumination. *Sattva* is described as: lightness, clarity, harmony, buoyance, illumination, lucidity, joy and understanding (Stoler-Miller, 2004; Bawra, 2012; Miller, 2012). The Bhagavad Gita highlights the importance of cultivating *sattva* as it is the foundation from which wisdom, discrimination and clear-seeing arises (Stoler-Miller, 2004). While *sattva* forms the base for many positive attributes, maladaptive states can also arise if one becomes overly attached to or dependent upon the quality of joy, as is briefly described in the Bhagavad Gita. In contemporary terminology words such as avoidance, unhealthy attachment, psychospiritual crisis or indifference have been used to describe this attempt to hold on to or maintain a static experience of *sattva* at the expense of allowing for the natural unfolding of the movement of the *gunas* within all BME and life experiences.

*Rajas* is the quality of energy, turbulence and pain that serves to activate. The quality of *rajas* is given a spectrum of emergent attributes that comes from this underlying capacity to mobilize and activate. On one end, *rajas* is said to support movement, creativity, motivation and activity. However, *rajas* can also underlie pain, anger, greed and agitation. The Bhagavad Gita explains that because rajas obscures knowledge and clear seeing, it impedes the yogi’s capacity to discern the difference between *prakriti* and *purusha*. *Rajas* balanced with *sattva* and *tamas* creates the motivation and creativity for inspiring change, movement and right action. Conversely, its preponderance may increase anger, agitation, or anxiety (Bawra, 2012; Miller, 2012).

*Tamas* is the quality of inertia, delusion and indifference that serves to restrain or limit. *Tamas* is explained through a spectrum of emergent attributes from this underlying capacity to restrain or limit. Theoretically, *tamas* may provide the support for experiences such as stillness, stability or groundedness. However, it may also foster dullness, inertia, obscuration, delusion, heaviness, negligence or ignorance. *Tamas* balanced with *sattva* and *rajas* may provide form and stability, whereas an over-predominance of *tamas* may give rise to delusion, inertia or obscuration (Bawra, 2012; Miller, 2012).

The Samkhya Karika offers the metaphor of a lamp to illustrate that all three *gunas* work together. Just as the wick, oil and flame work together for the purpose of illumination, the three *gunas* work together to reveal to the individual the difference between *purusha* and *prakriti* (Bawra, 2012; Miller, 2012; Larson and Īśvarakrsna, 2014).

The three *gunas* are in constant movement and co-existence as they co-mingle to create the various manifestations of BME phenomena (Bawra, 2012; Miller, 2012). The different proportions of *sattva*, *rajas* and *tamas* in each object of material nature, including the subtle mental components of personality, cognition, emotions and identity, give them their unique attributes (Stoler-Miller, 2004; Bawra, 2012; Miller, 2012). The movement and shifting nature of the *gunas* are intrinsic to life as they continually rise and fall, grow and diminish. Suffering arises from either trying to stop the movement of the *gunas* or from our relationship with each *guna*—not from the *guna* itself. Each of these qualities can hold positive attributes in their capacity to illuminate, activate, or restrain. However, our relationship to these qualities of material nature, and any attempt to maintain one at the expense of the others, may lead to imbalance, pain, or suffering.

Yoga teaches a methodology to nonjudgmentally and compassionately observe and experience the movement of the *gunas* such that the relationship and response to the changing phenomena of the BME is altered. The individual learns how to welcome and explore the BME in a way that facilitates eudaimonic well-being in the face of stressors or adversity.

## Convergence of Polyvagal Theory with the *Gunas*

Both PVT and the *gunas* provide a perspective to understand underlying foundations from which physical, psychological and behavioral attributes emerge. PVT provides insight into how underlying neural platforms are activated in response to perceived threat or safety in the presence of BME phenomena. Yoga suggests that physical, psychological and behavioral attributes emerge from and are influenced by the underlying interplay of the *gunas*.

Both frameworks discuss the co-existence and co-mingling of neural platforms (PVT) or *gunas* (Yoga) and attempt to convey complexity amidst an inherent tendency for reductionism within traditional academic disciplines. In PVT, the co-existence of neural platforms gives way to the varied experiences of play (safe mobilization) and intimacy (safe immobilization). In yoga, the co-existence of the *gunas* creates the varied phenomena of BME and influences the relationship and reaction to such stimuli. Both theories teach that it is from the surfacing of the neural platform of PVT or the *guna* of yoga that BME states are made manifest and established.

The *gunas* of yoga and neural platforms of PVT are also reflected in one another in a convergent and analogous manner. This relationship between the two models can be seen through the comparable descriptions of attributes. When the ANS comes under the influence of one of the *gunas* a distinct neural platform of the PVT may be activated supporting shared characteristics between the two. Likewise, when a neural platform is activated it supports the predominance of a *guna* and the shared characteristics between them emerge. For example, when *sattva* reflects through the nervous system the physiological, mental and behavioral characteristics of the VVC manifest, or when the VVC is activated the attributes of *sattva* manifest, as will be described in more detail below. This discussion explores the relationship between the two models in how they relate and affect one another for the emergence of physical, mental and behavioral attributes. Ultimately, this relationship is meant to foster an understanding that yoga therapy may affect both underlying neural platforms and *gunas*, resulting in improved self-regulation and resilience for the well-being of the individual.

## Comparative Look at Neural Platforms, Global States and *Gunas* (Table 1)

Both PVT and yoga describe three primary and combinable neural platforms or qualities from which specific physical, psychological and behavioral attributes emerge.

1. *Sattva* and the VVC: similar emergent attributes are found in descriptions of both *sattva* predominance and VVC activation. From *sattva* comes the realization of the connection between all beings. Sattvic joy is similar to eudaimonia with its more steadfast and everlasting quality and stems from calmness, tranquility and understanding of the “Self” within Stoler-Miller (2004). These attributes of connection, equanimity and eudaimonia are proposed to be dependent on a neurophysiological foundation for their emergence. Halifax proposed a model where qualities such as equanimity and eudaimonia emerge only when the system is sufficiently primed and includes an axis entitled “embodied/engaged” in which interoception is essential (Halifax, 2012). The VVC neural platform provides support for interoception, connection, equanimity and eudaimonia as it links awareness of bodily sensations with self-regulatory capacity; the use of facial cues and vocal prosody to communicate safety and connect to others; the tuning into human vocalizations to connect with others; the inhibition of defensive states to support equanimity, eudaimonia and connection through the capacity to nonjudgmentally listen, observe, and be in relationship with others (Porges, 2011, 2017). In sum through sattvic predominance and/or VVC activation the promotion of interoceptivity, connection, equanimity and eudaimonia may emerge. This state is well adapted for restoration, relaxation and connection and can be maladaptive when the individual is not able to adequately respond to the needs of the environment (including threat) as described within the other neural platforms and *guna* states.
2. *Rajas* and the SNS: the attributes that emerge from *rajas* and the SNS are shared in their spectrum from mobilization and activation to anger or fear. The *guna* of *rajas* and the neural platform of SNS provide a common foundation for activating and motivating forces. Similar to the state of safe mobilization and play which come when the VVC and SNS synergistically work together, when *rajas* co-arises with a balance of the other *gunas* attributes such as creativity, motivation, optimal action and change emerge. When *rajas* becomes predominant and is not balanced by the other two *gunas*, similar to the SNS, it provides the base for mobilization and movement in response to any demand for psychophysiological resources. This includes a spectrum from eustress to real or perceived threat in the environment with the emergence of behavioral attributes such as fear, anger, or aggression. This continuum of mobilization includes the well-adapted response to immediate threat, or may become maladaptive contributing to excessive allostatic load.
3. *Tamas* and the DVC: the attributes that emerge from *tamas* and the DVC provide a spectrum of experiences from stability and restraint to immobilization. In the same way the VVC and DVC partner to create internal conditions for the emergence of social bonding and intimacy, *tamas* can co-arise with a balance of the other *gunas* to manifest as stability and form. When *tamas* predominates, similar to the DVC neural platform, it provides the base for the emergence of obscuration, dullness, immobilization, inertia, or dissociation. This spectrum includes well-adapted responses to extreme threat or may become maladaptive and contribute to chronic disease states which has recently been proposed (Kolacz and Porges, in press).

**Table 1:** Characteristics and emergent properties of global states based on comparative neural platforms of Polyvagal Theory (PVT) and *Gunas* of yoga.

Global state	Neural platform	*Guna*	Emergent properties
Social engagement	VVC	*Sattva*	Safety, connection, clarity, eudaimonia, calmness, tranquility, equanimity
Play/Dance	VVC and SNS	*Rajas with Sattva and Tamas*	Activity, creativity, motivation, capacity for change
Fight or flight	SNS	Predominance of *rajas*	Fear, anger, greed
Intimacy	DVC with VVC	*Tamas with Sattva and Rajas*	Stability, form, restraint, social bonding
Shutdown/immobilization	DVC	Predominance of *tamas*	Obscuration, inertia, dullness, ignorance, delusion, dissociation

The parallels between PVT and *gunas* are further elucidated through the idea of the preparatory set. Five distinct preparatory sets stemming from PVT and the *gunas* are proposed with their integrated patterns of muscle tone/posture, autonomic state, affect, attention and expectation. As mentioned previously, by altering the underlying autonomic state, there are concomitant changes throughout the preparatory set. Analogously, an alteration in the predominance of a *guna* from which an individual is operating may create changes in the layers of the preparatory set from muscle tone/posture, autonomic state, affect, attention and expectation. This is congruent with the yoga therapy perspective, which utilizes an approach to evaluation and intervention that acknowledges the influence of the *gunas* on the physical, energetic, mental and behavioral aspects of the individual.

By including and emphasizing these underlying *gunas* and their effect on physical, psychological and behavioral attributes, yoga therapy can retain its integrated and comprehensive methodology based on its foundational teachings. The outcome of this understanding is a movement away from breaking apart the practices where *asana* (postures) are directed to musculoskeletal imbalances, *pranayama* (breathing practices) are directed to ANS state, and meditation or yama and niyama (intentional ethical principles) are directed to attentional, affective and cognitive states. A more cohesive approach can be implemented for both research and clinical applications where the evaluation, assessment and direction of intervention is toward affecting the underlying *guna*. The result would be an intervention consisting of yama/niyama, asana, pranayama and meditation given toward influencing the *gunas* and their correlated physical, mental and behavioral states. The resultant change in *gunas* would have concurrent effects on all layers of the preparatory set and on physical, psychological and behavioral well-being.

In sum, both PVT and *gunas* play a vital role in the understanding of how yoga may help diverse conditions and patient populations by affecting the underlying *gunas* and correlated neural platforms. Given the complexity of living systems, this integrative yogic approach to the whole person, while still necessarily reduced for explanatory purposes, has high potential for an effect on the emergence of integrated physical, mental, social and spiritual attributes and behaviors that facilitate well-being. These concepts will additionally support the yoga therapist in developing evaluation, assessment and intervention tools that are authentic to the foundations of yoga and the provision of its practices in a cohesive and comprehensive format while simultaneously assisting translation for researchers, the public and healthcare contexts.

## The Application of Yoga’s Model and Practices for Self-Regulation and Resilience

Yoga practices, when provided as a comprehensive methodology, are proposed to integrate autonomic, cognitive, affective and behavioral processes for regulation across physical, psychological and behavioral domains. Through both top-down and bottom-up practices, yoga may be effective at down-regulating the system towards parasympathetic, ventral vagal dominance (Streeter et al., 2012; Gard et al., 2014; Schmalzl et al., 2015). In addition, the application of yoga practices for resilience of the system will be discussed as they may support the individual’s capacity to work with shifting neural platforms and *gunas*.

Research has supported yoga’s benefit for diverse conditions such as depression, epilepsy, PTSD and chronic pain through its influence on the ANS and other inter-related systemic mind-body mechanisms which contribute to improved physical and mental regulation and decreased reactivity to stressful stimuli (Streeter et al., 2012). Research has also corroborated yoga’s effect on promoting vagal tone in diverse patient populations and its associated effects in decreasing allostatic load and enhancing self-regulation (Sarang and Telles, 2006; Khattab et al., 2007; Taylor et al., 2010; Telles et al., 2016; Tyagi and Cohen, 2016; Chu et al., 2017). In addition, yoga has demonstrated effect in being more than just physical exercise as it also concurrently benefits autonomic regulation, attention and affect (Mackenzie et al., 2014). Furthermore, research has supported yoga’s relationship to greater body awareness, compassion and eudaimonic well-being (Ivtzan and Papantoniou, 2014; Fiori et al., 2017). A program developed for adolescent depression included the goals of autonomic regulation, the practice of attentional and interoceptive awareness, and identification of intrinsic values to promote prosocial behavior based on yoga’s cohesive and integrative methodology and effect for regulation and resilience (Henje Blom et al., 2014).

Yoga is proposed to offer methods for regulation and resilience through the integrated practice of *yamas and niyamas* (ethical/intentional principles), *asana* (physical exercises), *pranayama* (*breathing techniques*) and meditation (Streeter et al., 2012; Gard et al., 2014; Schmalzl et al., 2015). Ethical intention setting (*yama and niyama)* informs and directs the meeting of physical and mental sensations, such as from interoception or emotions, for the promotion of positive physiological and affective states and prosocial behavioral responses (Gard et al., 2014). The ethical principles provided by yoga may help to guide the reaction, relationship and action of the individual in response to phenomena of the BME. For example, by meeting stimuli of the BME from a perspective of non-harming, non-attachment or contentment, the individual alters the way they pay attention to such stimuli, potentially facilitating an emergence of compassion, nonjudgment or acceptance (Gard et al., 2014). Just as Aristotle taught that virtue ethics provided guideposts for eudaimonia (Ostwald, 1962), the ethical principles of yoga elucidate a process to meet BME phenomena to facilitate the emergence of such qualities as eudaimonia. Asana, or physical postures may serve as a bottom-up regulatory tool to help regulate and promote resilience by altering the state of the ANS (Cottingham et al., 1988a,b; Schmalzl et al., 2015). Breathing techniques are known to directly affect cardiac vagal tone and the initiation of the vagal brake to move the system towards the VVC platform and provides another bottom-up regulatory practice of yoga (Brown and Gerbarg, 2005a,b; Porges, 2017; Porges and Carter, 2017) Finally, the yoga tradition offers an array of mind training practices for regulation, such as, focused attention and open monitoring meditation (Gard et al., 2014; Pascoe and Bauer, 2015; Schmalzl et al., 2015; Hofmann et al., 2016; Cramer et al., 2017).

## Yoga Practices for Self-Regulation: Facilitating VVC Neural Platform and *Sattva Guna*

As many of the beneficial characteristics for physical and mental health as well as prosocial behavioral attributes are shared by *sattva* and VVC, it is proposed that yoga practices may be utilized to strengthen one to affect the other. The VVC neural platform may be activated or made more accessible through practices that cultivate *sattva* and *sattva* may become more accessible or predominant through practices that activate the VVC neural platform.

The *Samkhya Karika* emphasizes the importance of fostering the quality of *sattva* through one’s habits, environment and behavior to realize the difference between *purusha* and *prakriti* and for the alleviation of suffering (Miller, 2012). This *sattvic* state is taught as being essential for the clarity needed to gather insight into the individuals relationship to various phenomena of the BME which may lead to healthy or unhealthy responses to stressors or stimuli (Stoler-Miller, 2004; Bawra, 2012; Miller, 2012). Through the clarity of *sattva guna*, the relationship to BME phenomena can be explored and healthy relationships to both interoceptive and outer stimuli can be cultivated.

Yoga therapy often first focuses on building a strong foundation of *sattva guna* to strengthen discriminative wisdom, develop mental clarity and increase systemic adaptability and resilience. Being established in *sattva guna* enables the opportunity to build positive internal relationships with interoceptive sensations, memories, emotions, thoughts and beliefs which may in turn support positive relationships with others. These features of *sattva guna* all benefit the self-regulatory capacity of the individual. From this *sattva guna* base the individual may experience the fluctuations of *rajas* and *tamas* and change their relationship and response to these qualities of material nature, potentially facilitating resilience of the system.

The VVC is a neural platform that supports physiological restoration, mental regulation and prosocial behavioral attributes. The VVC also provides a key anchor to build the critical self-regulatory skills that lead to greater systemic adaptability and resilience. Enhancing the individuals accessibility to VVC is proposed as a method to “retune” the ANS in disorders with a combination of diminished HRV as well as physical, mental and social health deterioration such as IBS and fibromyalgia (Kolacz and Porges, in press; Porges and Kolacz, in press).

Both VVC neural platform and *sattva guna* correlate with the emergence of such qualities as connection, tranquility, equanimity and eudaimonia. We propose that *sattva guna* shares neurophysiological features with VVC mediated states during which cardiac vagal tone is increased and the expanded integrated social engagement system is expressed. Both *sattva* and the VVC may be related to states of self-restoration, interoceptivity and the emergence of prosocial emotions and behaviors such as connection and eudaimonia.

As noted previously, yoga therapy’s explanatory framework can be described as the priming of the system for the emergence of eudaimonia with its concomitant physiological and mental health benefits (Sullivan et al., 2018). It is through the potential for eudaimonic well-being and shift in relationship to BME phenomena that yoga therapy is proposed to help with diverse clinical conditions and patient populations. Therefore, the reciprocal relationship between the VVC and *sattva*, which facilitates the emergence of eudaimonic well-being, is important to the yoga therapeutic process and application of yoga practices.

Building the individual’s facility with accessing and promoting *sattva guna* and the VVC neural platform is proposed to be a crucial and foundational step in learning of self-regulatory skills and from which resilience will emerge. Through self-regulation processes the nervous system enables healthy and more adaptive physiological, psychological and behavioral responses and provides opportunities for greater insight into the relationship to BME phenomena to lessen and alleviate personal suffering. Healthy adaptive physiological, psychological and prosocial states of eudaimonia, connectedness and equanimity may emerge when *sattva* and the VVC predominate. The practices of yama/niyama, asana, pranayama and meditation may enhance the function of the specific vagal pathways that optimize the neural platform of the VVC, and/or to strengthen the quality of *sattva guna* (Tsuji et al., 1994; Sarang and Telles, 2006; Telles et al., 2016; Tyagi and Cohen, 2016; Chu et al., 2017).

## Yoga’s Practices to Cultivate Resilience

Promoting practices that increase sattva or activation of the VVC can create a therapeutic container to safely challenge resilience-building. Within PVT, these challenges would be conceptualized as neural exercises expanding the capacity of the VVC to regulate state and to promote resilience. Yoga also includes various practices that achieve similar effects in optimizing autonomic control, providing greater physiological and psychological adaptability and resilience through reducing emotional reactivity and lowering the physiological set point of reactivity (Gard et al., 2014). In addition to developing down-regulating capacity, we believe meeting the needs of the environment requires the healthy navigation of VVC, SNS, DVC neural platforms and their combinations.

From a yoga perspective, the importance of resilience is also reflected in the model of the *gunas*. Just as the Bhagavad Gita elucidates the benefits of *sattva*, it also provides the ultimate aim of transcending the *gunas* through non-attachment, dis-identification and recognition of impermanence (Stoler-Miller, 2004). It is emphasized that this fluctuation and movement between clarity (*sattva*), activation (*rajas*) and restraint (*tamas*) is an intrinsic trait of all material nature (*prakriti*).

Since all behaviors and neurophysiological functions are dependent on movement of the *gunas*, the practice of yoga is not about staying in *sattva*, or limiting the movement of the *gunas*. Rather, yoga teaches a methodology to create a different relationship to the continual mixing and movement of these qualities. This underlying objective is shared with the neural exercise model of PVT. It is important to note that this state of discrimination between *Purusha* and *Prakriti*, or the *gunas*, is not one of non-participation or detachment from life. Instead, it is a state whereby the individual experiences the movement of the *gunas*, but the “world does not flee from him, nor does he flee from the world” (Stoler-Miller, 2004; p. 113). In other words, through yoga the individual learns not to ignore the movement inherent to BME phenomena, but to change the relationship with the movement of such phenomena. The practice is not meant to subjectively isolate oneself from the world, but provides a methodology and technology to experience the world such that suffering is lessened. PVT provides a neurophysiological explanation of the methods and techniques embedded in yoga.

Understanding and discriminating between this movement of the *gunas*, which make up the BME and that of “awareness”, or *purusha*, an individual is able to experience deep equanimity, inexhaustible joy, and a sense of pure calmness even when the movement of the *gunas* continue (Stoler-Miller, 2004). When one learns to nonjudgmentally observe and experience this movement of the *gunas* an unwavering and profound capacity for equanimity and eudaimonic joy emerges (Figure 1). The individual changes their relationship with the fluctuations of the BME and learns to respond and receive the changing phenomena of life differently.

The practices of yoga may serve in this development of resilience through the idea of safe mobilization and safe immobilization. Just as in the state of safe mobilization, there is activation of the SNS within a container of the VVC for safe activation of the system. Similarly, within a foundational platform of *sattva* the individual is able to utilize the rising of *rajas* for creativity, motivation or change, rather than *rajas* becoming a negative force. By developing an improved ability to recruit and engage the neural platform of VVC or *sattva*, there is greater resilience when confronted with disturbances. For example, the individual can learn techniques of ethical intentional setting, attentional control, various other meditations, breath and movement to cultivate *sattva* and maintain the neural platform regulated by the VVC. Then the individual can assume challenging or activating postures or breath techniques that mimic the activation of the system. Resilience is cultivated by maintaining or building the facility to find calm mental or physiologic states while activated. The individual is able to learn to move between *guna* states and neural platforms and/or to experience the combined state of *sattva* with *rajas*, or neurophysiologically promoting a neural platform that integrates VVC with SNS.

By working with the qualities of *rajas* and *tamas*, while maintaining access to *sattva*, the window to tolerance for sensation may be widened and resilience facilitated. Through maintaining the neural platform of the VVC while activating the system, as well as alternating between relaxing and activating practices, the individual may foster both regulation and resilience of the system. Thus, through the maintenance of the foundation of *sattva*, which provides clarity and insight while experiencing activation, the individual may find ways to change the relationship to BME thereby learning tools of self-regulation that enhance resilience. The capacity to discern, alter reactivity, and even to hold a positive attitude in the presence of activation offers an important resource in promoting self-regulation, which may be utilized in response to stressors in the BME including the experience of pain, illness or disability and thereby also improving resilience both physiologically and psychologically.

The yoga therapy process encourages a foundation of safety/VVC from which rajas/SNS and tamas/DVC can be experienced with greater adaptability and resilience with the broad relationship to BME phenomena (Figure 1).

## Discussion

This article offers a theoretical model based on a convergent view of yoga and PVT; two analogous explanatory systems for understanding the function and interplay of underlying neural platforms (PVT) and *gunas* (yoga), and their role in manifesting physiological, psychological and behavioral attributes. By affecting the neural platform, or *guna* predominance and relationship to these shifting neural platforms, or qualities, the preparatory set of the individual is altered. The development of interoceptive awareness and sensitivity fosters regulation and resilience to these shifting neural platforms and *gunas* in response to phenomena of the BME. In addition, the *gunas* of yoga and the neural platforms of PVT share characteristics that parallel one another where the neural platform reflects the *guna* predominance and the *guna* predominance reflects the neural platform.

Yoga is suggested to provide a form of neural exercise, and a methodology of working with the *gunas*, for the regulation and resilience of the system. Through altering and/or changing the conscious relationship to underlying state of the *gunas* and neural platforms described by PVT, the preparatory set is affected and physiological, psychological and behavioral processes are reciprocally influenced (Payne and Crane-Godreau, 2015; Porges and Carter, 2017). Yoga practices may promote the accessibility of the VVC and the relative balance of *sattva* to the other *gunas* to assist processes of physiological restoration and positive psychological and behavioral states. Yoga practices may also be seen as helping develop facility with moving in and out of relative dominance of these theoretical neural platforms and *guna* states such that resilience of the system is cultivated. As an individual learns tools for self-regulation to explore and potentially alter the relationship to BME phenomena, the relationship to suffering may be improved (Figure 1).

While we do not wish to convey that the end-goal is cultivation of *sattva*, the theoretical correlation to the neural platform of VVC may be seen as a neurophysiological substrate or stepping stone towards the emergence of such states as eudaimonia, connection, or tranquility. Similarly, we are not suggesting that the other *gunas* and neural platforms are “bad” as these energies and states are inseparable and adaptive in understanding the complexity of human experience and behavior, and thus the potential influence of a yoga therapy framework for well-being. The capacity to cultivate eudaimonic well-being is significant to yoga therapy’s explanatory framework in benefiting diverse patient populations and conditions for physical, mental and behavioral health and well-being. The states of eudaimonia, calm, or tranquility that may emerge from the cultivation of yoga practices influences the preparatory set such that healthier relationship to BME conditions may be learned and self-regulatory skills can be built.

From a strengthened foundation of the neural platform of VVC, and the *guna* of *sattva*, the individual has the resources to move through states of dominant *rajas* and *tamas* or SNS and DVC such that adaptability, flexibility and resilience of the system is cultivated. The practices of yoga that engage both top-down and bottom up processes may be utilized for the cultivation of resilience by moving between neural platforms and *gunas* (Figure 1).

This model may be utilized in several ways. In one practice of this model, *sattva* and the VVC serve as methods for self-regulation and the practices of yoga build the tools to return to and strengthen these restorative and calm states. This is a useful practice if an individual experiences maladaptive or overwhelming states of *rajas* and SNS, or of *tamas* and DVC, and can learn to utilize yoga therapy techniques to return to *sattva* or VVC for clarity and calm. In another practice of this model, the individual learns to change the relationship with and widen their threshold of tolerance to both *rajas* and *tamas*, and SNS and DVC, thereby increasing the experience of safe mobilization and safe immobilization. This means that the individual learns how to find support in an underlying *sattva* or VVC state while other platforms or qualities of *rajas* and SNS, or *tamas* and DVC, are activated. An example is the utilization of postures or breath techniques that activate the system, while simultaneously engaging in practices of intentions, meditations, and breath techniques to facilitate the underlying sense of connection, calm or tranquility.

The parallel between affecting the underlying neural platform or *guna* state is significant in enabling yoga therapy to be practiced in a manner consistent with its philosophical foundations while being translatable to current neurophysiological thoughts. The ability to utilize the existing explanatory framework provided by yoga and the gunas combined with the biobehavioral model established by PVT enables the translation of yoga therapy into healthcare and research without the need to adopt an outside neurophysiological model and attempt to fit yoga into that model.

This work will contribute to yoga therapy being understood as a distinct healthcare profession which benefits physiological, psychological and behavioral well-being for diverse patient populations through the cultivation of self-regulatory skills, resilience and eudaimonic well-being. This model provides support for the creation of yoga therapy assessment tools to identify underlying *guna* predominance. In addition it supports the yoga therapist in evaluating, assessing and creating interventions aimed at working with underlying *guna* predominance and identifiable and measureable neural platforms towards these goals. Rather than creating protocols for allopathic conditions, the yoga therapist influences these underlying *guna* states and neural platforms to target the unique needs of each individual. Bridge building efforts such as this work would support yoga therapists, who work with clients in a manner that is simultaneously based on yoga foundational theory and offers additional translatory language for research, the public, and integration into healthcare.

## Implications and Future Directions

The theoretical model put forth in this article points to several implications and future directions.

It is suggested that yoga therapy research include the comprehensive system of yoga in intervention protocols including: yama and niyama (ethical intentional practices), asana (postures), pranayama (breathing practices) and meditation.

In order to reflect the intention of yoga, the application and research of yoga for diverse populations would benefit from being directed toward such ideas as facilitating eudaimonic well-being. In addition, the targeting of yoga therapy interventions to underlying *guna* states or neural platforms to enhance self-regulation and resilience and its relationship to the cultivation of eudaimonic well-being is proposed.

Several directions of future study are proposed:

a. Examining eudaimonic well-being, interoception, and indices of HRV (e.g., respiratory sinus arrhythmia) as underlying mechanisms through which yoga therapy improves outcome measures such as: improved quality of life, self-regulation, resilience and decreased pain, inflammation, perceived loneliness, anxiety and depression in diverse patient populations and conditions.
b. Exploring the relationship between indices of HRV, measures of interoception, eudaimonic well-being and their connection to physical, psychological and behavioral health and well-being both quantitatively and qualitatively.
c. Testing the hypothesis of the convergence of neural platforms and *gunas*. It would be of particular interest to investigate whether the physiological states identified by PVT parallel the “states” and processes described in yoga. For example: Is the VVC state, expressed as increased respiratory sinus arrhythmia, decreased blood pressure and decreased catecholamines, associated with subjective experiences such as calm, equanimity and connection that practitioners describe as *sattva?* Is the SNS state, expressed as decreased respiratory sinus arrhythmia, increased blood pressure and increased catecholamines, associated with subjective experiences such as anxiety, fear or worry that practitioners describe as *rajas?* Is the DVC state, expressed as decreased activity, decreased heart rate and decreased blood pressure, associated with subjective experiences such as a disconnect from the world that practitioners describe as tamas?
d. Continued definition of the *gunas* and relationship to the neural platforms and the utilization of their assessment, evaluation and targeted intervention to facilitate regulation, resilience and physiological, psychological and behavioral health and well-being in diverse client populations and conditions.

## Conclusion

Yoga therapy is proposed to facilitate eudaimonic well-being with its many effects for physical, mental and behavioral health for diverse populations through the building of self-regulatory skills and cultivating resilience of the system (Figure 1). The attributes of the *gunas* of yoga and the neural platforms of the PVT, while not the same, are reflected in one another. As such, working with *gunas* and neural platforms that underlie physical, psychological and behavioral attributes, provide a methodology for the application of yoga practices for facilitating systemic regulation and resilience.

Yoga therapy builds a strong foundation in *sattva* and the neural platform of the VVC for the emergence of connection, tranquility and eudaimonia with resulting benefits to physiological, psychological and behavioral health and well-being. In addition, resilience is facilitated through changing the relationship to the natural fluctuations of the *gunas* of *rajas* and *tamas*, and their counterpart neural platforms of SNS and DVC, such that the individual learns to effectively “bounce back” to states of restoration and build resilience (Figure 1).

It is when yoga is practiced and understood as a cohesive and comprehensive system that the benefits for self-regulation and resilience may be realized. As one learns new responses to potential BME stressors, he or she may experience greater physiological, psychological and behavioral health and well-being. The convergence of PVT and the *gunas* may help frame yoga therapy as a method that supports self-regulation, resilience, and for lessening allostatic load through building healthy relationships to BME phenomena. When yoga therapy is applied through this perspective of shifting underlying *guna* states and neural platforms, the integrated nature of the practice can be understood as distinct from other CIH practices. It is hoped that this helps inform both research and healthcare contexts interested in integrating yoga interventions for various patient populations and conditions.

## Author Contributions

All authors contributed to theoretical discussions as well as the writing of this manuscript.

## Conflict of Interest Statement

The authors declare that the research was conducted in the absence of any commercial or financial relationships that could be construed as a potential conflict of interest.

## Footnotes

^1^www.IAYT.org
